# Implementing the “Getting It Right First Time” (GIRFT) Report Recommendations: The Results of Introducing a Shoulder and Elbow Multidisciplinary Team

**DOI:** 10.7759/cureus.23338

**Published:** 2022-03-20

**Authors:** Hammad Parwaiz, Robert Whitham, Matthew Flintoftburt, Andrew Tasker, David Woods

**Affiliations:** 1 Trauma and Orthopaedics, Great Western Hospital, Swindon, GBR

**Keywords:** mdt, elbow, shoulder, girft, getting it right first time

## Abstract

Objective

In this study, we aimed to analyse the impact of implementing the “Getting It Right First Time” (GIRFT) recommendations in our shoulder and elbow unit, which included the introduction of a shoulder and elbow multidisciplinary team (MDT) meeting for all patients being considered for surgery.

Methods

A retrospective patient case-note review was undertaken to assess the impact of replacing the pre-admission clinic with an MDT meeting. We analysed how many of the proposed management plans were changed as a result of this new MDT, as well as the associated cost savings.

Results

Of note, 118/148 patients who attended the MDT had a provisional operative plan; 24/118 (20%) had their plan changed to non-operative management, 13/118 (11%) had a change of operation, and 6/118 (5%) were recommended further investigations or tertiary referral. This reduced theatre time required by 47 hours, an estimated saving of over £51,000. Significantly, 20/24 patients who had their plan changed from operative to non-operative still had not had an operation after a median follow-up of 39 months.

Conclusion

The introduction of a shoulder and elbow MDT for all patients being considered for an operation has improved decision-making, allowed optimisation of non-operative management, and helped prevent patients from having unnecessary operations. This has led to a better patient experience and a more efficient service delivery, which is associated with cost savings.

## Introduction

In this age of financial austerity, publicly funded services in the UK are under extreme pressure to cut costs and enhance efficiencies while maintaining the same quality of service delivery. The National Health Service (NHS) is no exception to this. With an ageing population, demand for healthcare services is going to increase considerably over the next few decades.

The “Getting It Right First Time” (GIRFT) report was first published in 2012 [1] and examined the current state of orthopaedics in England in terms of pathways of care, patient experience, and outcomes. It set out to improve the quality of care, reduce complications and thereby save significant sums of money. Since then, the Department of Health and NHS England have funded a national pilot of the GIRFT project with the aim of improving medical care within the NHS by reducing unwarranted variations. It focused on elective adult orthopaedic and spinal services in England, involving over 220 hospitals across the country. It examined clinical outcomes, processes, patient pathways, network arrangements, and financial impacts. There were significant variations in practice and outcomes, including infection, readmission, and return to theatre rates, the volume of activity within each department for certain procedures, and costs of prostheses, especially when procured as loan kits [2].

In the wake of this report, our unit was visited by the GIRFT team. Among the national recommendations, we had already put in place methicillin-resistant *Staphylococcus aureus* (MRSA)-protected wards and specialist theatre teams. A number of further recommendations were made, including (a) the introduction of a shoulder and elbow multidisciplinary team (MDT) meeting; (b) dual consultant operating for difficult cases; (c) referral of infrequently done operations to a specialist centre (in our case, total elbow replacements); and (d) careful procurement of prostheses.

This article analyses the impact of the introduction of a new shoulder and elbow MDT, and explores the benefits conferred.

## Materials and methods

We introduced a weekly MDT meeting and merged this with our pre-existing preoperative assessment clinic (PAC), which had formerly been handled by a lone consultant. These were attended by two consultants, specialist physiotherapists, pre-assessment nursing staff, as well as our associate specialist and trainees when available.

Prior to this, patients who were referred to our unit with a shoulder or elbow problem were triaged and seen by either a trained extended scope physiotherapist (ESP) in our Musculoskeletal Assessment and Treatment Service (MATS), an orthopaedic specialist doctor working independently, by one of the two consultant shoulder and elbow surgeons, or their supervised registrar. Day cases were listed and, provided they were medically fit, would not be seen again until the day of surgery. Those patients who were listed for more complex surgeries requiring an inpatient stay would attend the PAC four to six weeks prior to their operation.

We changed this system to bring all patients listed by non-consultant grades, those requiring inpatient stay, and any consultant-listed case warranting further discussion to the MDT. The meetings only occurred on those weeks when both consultants and at least one ESP were available. This only applied to our elective workload.

Data collection and statistical analysis

An analysis of all patients presenting to the upper-limb MDT in 2017 was performed. We recorded the initial diagnosis, the operation proposed, the grade of staff proposing the surgery, and whether the plan was altered by the MDT meeting.

The amount of theatre time allocated for each procedure was recorded. We were therefore able to estimate both the amount of theatre time and the cost to the trust incurred by the changes made at the MDT. The NHS Institute for Innovation and Improvement estimates hourly theatre costs at £1,200 [3]. The allocated time for each case in the theatre is listed in Table 1. The hourly costs of consultant (£46.88), associate specialist (£38.15), band 7 physiotherapist (£21.37), and band 5 nurse time (£14.70) were provided by our payroll department.

**Table 1:** Allocated theatre time for individual procedures

Procedure	Allocated theatre time (minutes)
Reverse total shoulder replacement	120
Anatomical total shoulder replacement	120
Latarjet procedure	120
Arthroscopic shoulder stabilisation	90
Subacromial decompression and cuff repair	90
Subacromial decompression	60
Ulnar nerve release	45
Tennis elbow release	30
Manipulation of the shoulder under anaesthesia	15

We subsequently reviewed the outcomes of those patients who had their management changed from operative to non-operative to see whether they ultimately ended up having an operation at a later date. The MDT was only for the discussion of those cases that the referring clinician felt may benefit from surgery; therefore, no patient had a change of treatment from non-operative to operative.

## Results

We held 31 MDT meetings in 2017, attended by 148 patients (mean: 4.8 patients per clinic). The clinics took approximately two hours. They were attended by both consultants, and 1.4 physiotherapists on average. The associate specialist and trainee registrar (SpR) were supernumerary, and hence no clinical activity was lost by their attendance. The previous PAC clinics were handled by a single consultant, on average 40 weeks per year; they took 1.5 hours and were not attended by a physiotherapist.

**Figure 1:**
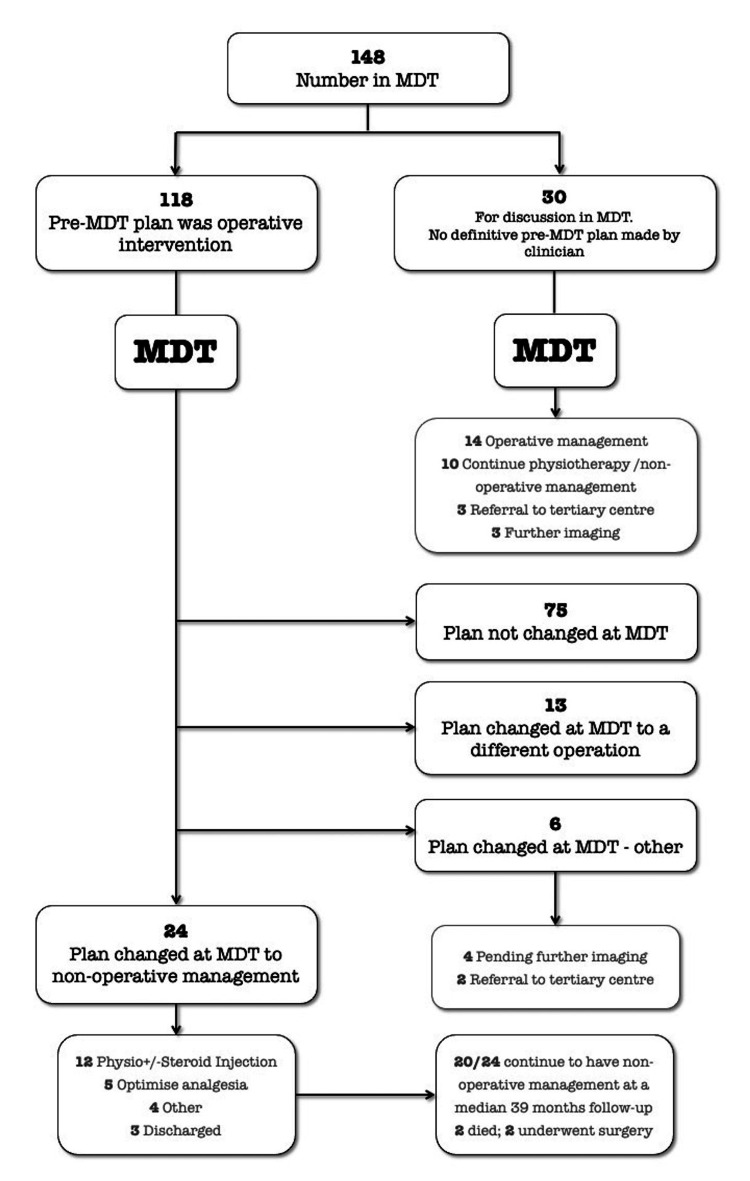
The flowchart showing the decision-making process for all 148 patients who went through the shoulder and elbow MDT meeting MDT: multidisciplinary team

Of note, 118/148 patients attending the MDT had an initial operative plan, with 30/148 having no definitive pre-MDT plan made by the referring clinician (Figure 1); 24/118 (20%) patients had their initial operative plan changed at the meeting to non-operative treatment, 13/118 (11%) patients had a change to the proposed operation, and a further 6/118 (5%) were referred for further imaging or to a tertiary centre, leading to a total of 43/148 (36%) having a change in their plan after MDT. And 5/148 (3.4%) patients had a change in their analgesia regime; 22/148 (15%) patients had a course of physiotherapy ± a cortisone injection.

After a median follow-up of 39 months after the MDT, 20/24 patients were still in the non-operative group. Two died and two subsequently underwent surgery at 21 and 33 months post-MDT. Comparing all referrals made by clinicians of each grade, no differences were observed in outcomes between consultants, physiotherapists, and middle grades, with approximately two-thirds of all referrals remaining unchanged and the other third being changed or sent for further imaging or tertiary referral (Table 2). A comparison of the SpR group was difficult due to low numbers.

**Table 2:** Outcomes of referrals to MDT by clinicians of different grades MDT: multidisciplinary team

Clinician making referral	N	MDT outcome	N	%
Consultant	70	Same plan	35	50
		Plan changed	15	21
		Further investigation/tertiary referral	6	9
		No plan prior to MDT	14	20
Physiotherapist	46	Same plan	25	54
		Plan changed	11	24
		Further investigation/tertiary referral	4	9
		No plan prior to MDT	6	13
Middle grade	24	Same plan	13	54
		Plan changed	9	38
		Further investigation/tertiary referral	1	4
		No plan prior to MDT	1	4
Speciality registrar	6	Same plan	1	17
		Plan changed	2	33
		Further investigation/tertiary referral	1	17
		No plan prior to MDT	2	33

A total of 42 patients were referred to the MDT with an initial plan for subacromial decompression (SAD): the most common procedure to have the plan changed; 20 of these plans were changed at MDT: nine were sent for specialist physiotherapy ± steroid injection, five for manipulation under anaesthesia (MUA), two for further imaging, two were discharged, one was sent for hydrodilatation due to a diagnosis of frozen shoulder, and one for neurology review as the pain did not originate from the shoulder.

After SAD, reverse geometry total shoulder replacement (RTSR) was the next most common procedure to be changed at MDT. Of the 29 proposed RTSRs, five required optimisation of analgesia, two were advised to have a less invasive procedure, one required further imaging, and one was referred to a tertiary centre for a second opinion.

Cost of implementing an MDT

**Figure 2:**
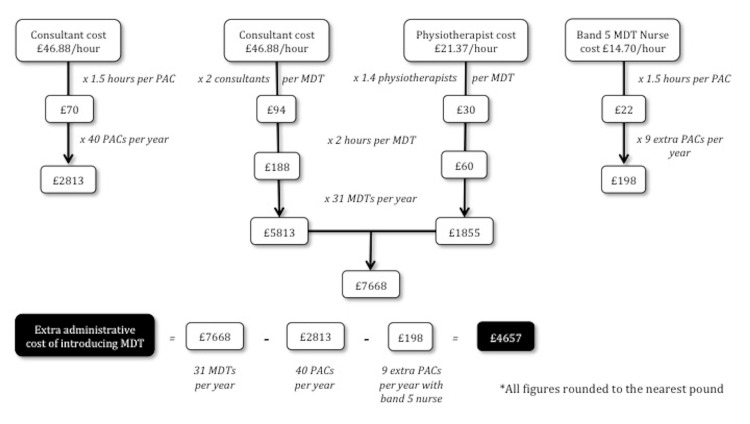
The flowchart showing the costs of implementing a preoperative assessment clinic (PAC) versus a multidisciplinary team (MDT) meeting These calculations show that the administrative costs of implementing an MDT are slightly higher compared to a PAC

The cost of implementing 31 MDT clinics was £7,668 while it was £2,813 for 40 PACs. As there were nine fewer MDTs than PACs per year, a saving of £198 per annum for the band 5 MDT nurse was achieved. Combining these costs, the introduction of an MDT cost an extra £4,657 per annum (Figure 2).

We calculated the theatre time taken for each planned operation, including anaesthetic time (Table 1). A total of 43 patients had their plan changed at MDT, including 24 to non-operative management. This led to 47 hours of theatre time being saved, which in turn equates to savings of £56,400 per annum, and taking into account the extra administrative cost of implementing an MDT, there was an overall saving of £51,743 per annum.

## Discussion

In the wake of the 2015 GIRFT report on orthopaedic services in the UK, several changes have occurred in the field of orthopaedic practice nationally. Despite this, there is a paucity of literature on outcomes from individual trusts and departments, and this makes our paper one of the first attempts to provide some data on this topic. The GIRFT study group has recently published a study protocol for the next phase in their project: to examine whether the planned changes have delivered improvements in the quality of care and patient outcomes [4]. This paper shows the evidence of its delivery based on our experience.

Although an MDT is costly in terms of the time spent by all members of the team, it produces a more tailored treatment plan, which the patients appreciate. It could be argued that we could have made several changes to the plan with a routine PAC itself, e.g., regarding those not fit for surgery or when the patient opts to be treated conservatively; however, having ESPs who can offer the patient specialist physiotherapy care present is a very appealing option for the patient. There may be situations where an ESP can examine the patient in the MDT, and there is much benefit in providing focused physiotherapy that a surgeon working in isolation would not be able to appreciate. There have certainly been benefits from reviewing all the proposed day cases, who often had the plans changed at the MDT due to change of circumstances or diagnosis, or because of a more considered opinion.

Crucially, in a system where multiple individuals are listing patients for surgery, an MDT acts as quality control and is a learning opportunity for all involved, especially the ESPs and surgical trainees to better understand the process of surgical decision-making from senior clinicians. Conversely, it also allows surgeons to appreciate the scope of specialist-focused physiotherapy in mitigating the need for surgery.

We have had a high threshold for recommending surgery, particularly for patients with impingement, for whom steroid injections and physiotherapy is our default treatment. Furthermore, a recent re-visit by the GIRFT team commended the department in light of the findings of a recent report into the volume of individual upper-limb operations within each hospital [5]. The GIRFT report mentioned a “746% increase in the number of patients undergoing arthroscopic subacromial decompression in the last 10 years with no long-term data on outcomes,” describing it as a procedure with “little evidence of clinical efficacy.” Our unit was found to be over two standard deviations below the national average for this particular procedure and our results showed that 20/42 patients referred to MDT with an initial plan for SAD had their plan changed.

Beard et al. in the CSAW trial, a large multicentre randomised control trial comparing decompression versus arthroscopy alone versus no treatment for SAD, found no benefit in arthroscopic SAD over arthroscopy alone, with the observed difference between the surgical and non-surgical treatment groups being attributed to postoperative physiotherapy provided in those groups [6]. Although some smaller case series suggest that there is benefit and cost-effectiveness in performing this procedure in carefully selected patients [7], most studies have shown little benefit compared to physiotherapy or no treatment [8-10]. Furthermore, specific physiotherapy procedures focused on the rotator cuff and scapular stabilisers have been shown to have significant benefits in treating subacromial impingement [11-12].

The benefits of surgery versus physiotherapy or other non-operative treatment have also been questioned with regard to other common shoulder pathologies, such as frozen shoulder [13-16], and hence the involvement of physiotherapists in our MDT is crucial. Furthermore, the involvement of different professions in patient care can help improve patient satisfaction [17].

The main reasons for changing the operation

(1) Patients who initially had a diagnosis of impingement and had not responded to a cortisone injection and physiotherapy were often put on the list for a SAD. At the MDT, it became apparent that they had developed a frozen shoulder and the operation was therefore changed to an MUA.

(2) Patients had had inadequate analgesia or physiotherapy, and hence an improved analgesia regime or specialist physiotherapy was implemented.

(3) Patients who were listed for surgery had a greater opportunity to discuss their operation and other options in more detail and received a balanced view from different members of the MDT.

Finally, the calculations do not include the savings to the NHS achieved by reducing the complication rates, the time taken for cancelled operations on the day, and the administrative costs of fielding complaints and rescheduling patients.

Limitations

While we have been able to show the benefits of introducing a shoulder and elbow MDT, this approach has to be tailored for each unit or department planning to implement its own MDT. Work plans have to be reconfigured to have two consultants and all other members of the MDT to be present, and this requires support from department managers. In trying to replicate the success of our MDT, we anticipate each unit to face its own unique challenges, the discussions of which are beyond the scope of this article. We hope, however, that our experiences described in this article can act as a template for others.

We have estimated our cost savings based on theatre time saved, and the cost of employing each member of the MDT. In reality, the healthcare system is far more complex and there may be some costs that we have not been able to factor in. For example, although significant sums of money would be saved from avoiding an operation (cost of theatre time, staff costs), there would still be costs of further imaging, nursing care, and physiotherapy sessions for these patients, albeit far less. It could, however, be argued that these costs ultimately cancel out, as every surgical patient would have to have postoperative follow-up and potentially further physiotherapy. Surgery in itself is not the end of the treatment pathway for these patients.

Finally, future work could consider examining patient-reported outcome measures or conducting patient surveys to assess the patient experience. To some extent, in the literature, this has been explored for individual pathologies such as subacromial impingement and frozen shoulder (as referenced earlier), but devising a specific way to measure patient satisfaction in our new MDT pathway could be the next step.

## Conclusions

Implementing the MDT process as described in this paper and as recommended by the GIRFT report has led to better patient care, optimised treatment plans, reduction in the number of patients undergoing unnecessary surgery, and significant financial savings for the shoulder and elbow team in our department. It has led to additional benefits such as opportunities for team building and continued professional development of the whole team. Even though we were initially skeptical about the practicality of adding these changes to an already busy schedule, we have been persuaded that these changes have resulted in significant improvements in our service at a decreased cost to the taxpayer. We believe that these simple changes can easily be replicated in other units.
